# HoloVAD; A feasibility study of a new patient education tool for patients with a left ventricular assist device

**DOI:** 10.1016/j.pecinn.2025.100450

**Published:** 2025-12-06

**Authors:** Sofie E. Bresser, Linda M. de Heer, Irene Louwerse-van Kan, Faiz Ramjankhan, Manon G. van der Meer, Saskia W.M. Weldam

**Affiliations:** Institution: Division of Heart and lungs, University Medical Centre Utrecht, Utrecht, the Netherlands

**Keywords:** Patient education, Mixed reality, Left ventricular assist device, Feasibility studies

## Abstract

**Objective:**

Patient education for patients with a left ventricular assist device (LVAD) and their loved ones is crucial for improving adherence and reducing complications. HoloVAD is an innovative virtual education tool, which offers a preimplantation experience of life with an LVAD (Holomoves ©, The Netherlands). Our objective is *to* assess the feasibility, defined as acceptability, usability, and tolerability, of HoloVAD.

**Method:**

A cross-sectional, quantitative feasibility study was conducted among patients with an LVAD, and their loved ones.

**Results:**

In total 45 participants were included. Six out of the seven acceptability domains of the Unified Theory of Acceptance (UTAUT) were scored positively. With an excellent overall SUS-score (82.5) the HoloVAD application is assessed as usable for both groups, and tolerability is scored as positive at every point of the Virtual Reality Sickness Questionnaire (VRSQ), for both groups.

**Conclusion:**

HoloVAD is feasible - defined as being acceptable, usable, and tolerable -and represents a promising and helpful new education tool for the study population.

**Innovation:**

An LVAD is life-changing with high complication risks. HoloVAD is innovative because it integrates visualization and interaction through mixed reality, enabling a deeper understanding of life with an LVAD. This study highlights the feasibility of HoloVAD as a novel educational tool for patients and their loved ones.

## Highlights

• Using mixed reality in patient education is a beneficial technique.
• HoloVAD allows patients to explore life with an LVAD before implantation.
• Preimplementation experience has many benefits for the quality of patient care.
• HoloVAD shows high acceptability, usability and tolerability among participants.
• HoloVAD is feasible for providing preoperative patient education.

## 1 Introduction

Heart failure (HF) affects 64 million people worldwide and while pharmacotherapy and lifestyle changes manage most HF patients, severe cases could require interventions such as left ventricular assist device (LVAD) implantation or heart transplantation [1,2]. Living with an LVAD, a mechanical pump used to support left ventricular function, involves many responsibilities and changes in daily routine. Additionally, patients undergoing general anesthesia and major surgery often experience stress and anxiety [3,4] These factors can cause burdens and stress, related to handling the device itself and psychosocial aspects of life [2,5]. [3,4]. Offering patients and their loved ones information about their hospital stay, anesthesia, the operation, the LVAD, and life after implementation helps patients understand what to expect, improves treatment adherence, and reduces their worries and anxieties [6,7]. Education about the expectations of living with an LVAD, can help patients manage their expectations and improve their ability to cope [5,8]. Therefore, effective good quality patient education is important for LVAD patients, and their loved ones and further research is needed to develop interventions, particularly educational interventions for better adaptation for LVAD patients [5,8].

Effective patient education yields numerous advantages for LVAD patients and their loved ones, concerning quality of life (QoL) and complication management [5,8]. Patient education is the process of influencing patient behavior and producing changes in the knowledge, attitudes, and skills necessary to maintain or improve health [9]. It is integral to health care quality and must be patient centered, straightforward, and multimodal [10]. Multimodal education, which engages multiple senses during the learning process, improves long-term and working memory [11,12]. Effective education also requires stimulating both cognitive and affective learning. [13,14]. Additionally, interactivity and the involvement of different senses should be incorporated to enhance information retention [15].

**Fig. 1:**
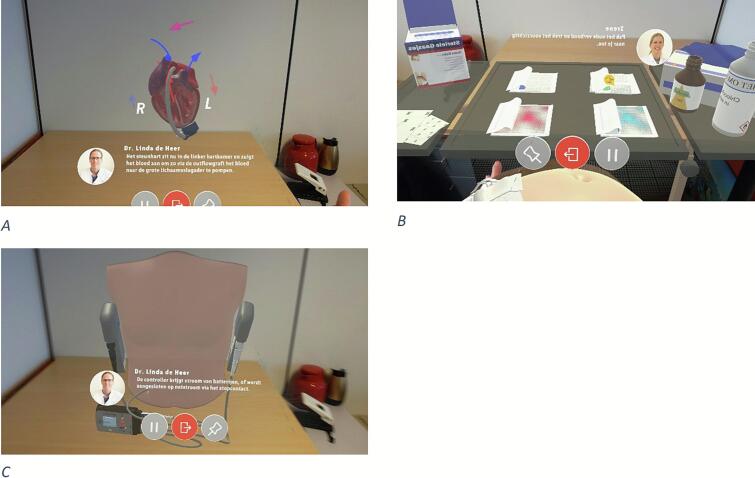
Screenshot of images of the HoloVAD (copyright; UMC Utrecht, The Netherland). A; a thoracic surgeon explaining about heart failure and B; a nurse specialist explaining how to do the wound care. C; a thoracic surgeon explain about a Left Ventricular Assist device.

Aspects for effective patient education can be facilitated through innovative techniques such as mixed reality (MR). MR enriches the real world with interactive virtual data anchored independently of head movement [16]. MR can be experienced through a HoloLens, a mixed reality head-mounted display (HMD), that overlays virtual objects onto the real world [17,18]. Other virtual tools, for other virtual worlds, have been proven effective for educating both professionals and patients [19,20]. MR has been proven to be beneficial for the education of professionals in the surgical field [16,21] and is suggested to be beneficial for patient education [16]. Research has shown that the use of MR can improve patient understanding and decrease anxiety, and as mentioned above, MR could have many benefits for LVAD patients [16,22]. To our knowledge, MR patient education has not been tested in LVAD patients, and further research on the development of educational innovations for this vulnerable population is needed. For this next step in patient education for patients receiving an LVAD, we developed HoloVAD in collaboration with Holomoves, a mixed reality company from Utrecht, the Netherlands, that is involved in the development of applications for rehabilitation. This mixed realty application has various educational and interactive modules related to an LVAD (see Fig. 1). In contrast to existing MR tools, which are generally designed for broad rehabilitation purposes, HoloVAD is uniquely tailored to patients with an LVAD, providing disease-specific education and interactive modules that directly address the challenges of living with this device.

HoloVAD was developed to educate patients, and their loved ones, about living with an LVAD and its challenges using MR, with the goal of improving expectations, QoL and complication management.

HoloVAD constitutes a complex intervention for patient education. According to the Medical Research Council (MRC), the feasibility of new complex interventions should be evaluated [23]. This step is essential for assessing the practical implementation of HoloVAD before broader deployment. Feasibility evaluation enables the identification of potential barriers, refinement of HoloVAD design, and determination of the resources required for a larger-scale trial. Given that HoloVAD has been developed and shows promise for routine application, conducting a feasibility study is logical for the next step [23]. Therefore, the primary objective of this study was to assess the acceptability, usability, and tolerability of HoloVAD for patients with an LVAD. A secondary objective is to assess the acceptability, usability, and tolerability of HoloVAD for loved ones of these patients and to compare these findings with those obtained from the patients themselves.

## 2 Method

### 2.1 Design and context

This study employed a cross-sectional, quantitative design to assess feasibility among patients with an LVAD—treated at a university hospital in the Netherlands—and their loved ones. In this article, the study population refers to patients with an LVAD and their associated loved ones. Recruitment and data collection took place between January 2024 and April 2024.

Although the HoloVAD modules were ultimately developed for preoperative education, this feasibility study focused on postoperative patients. This decision was made because of ethical considerations. Postoperative patients are less vulnerable than preoperative patients are, allowing for an initial assessment of their experiences with the modules and an evaluation of the feasibility of HoloVAD [23]. Feasibility was assessed using acceptability, usability, and tolerability as outcome measures.

Patients treated at the outpatient clinic of the included university hospital were recruited using a consecutive sampling approach. Approached patients were invited to bring a loved one but could also participate alone. Eligible participants were required to have sufficient arm function to operate the HoloLens device and adequate proficiency in Dutch to follow and engage with the HoloVAD modules. Participants were excluded if they were ≤ 18 years old or unable to complete a Dutch-language questionnaire because of or mental cognitive impairments or because of a language barrier. Data collection and reporting adhered to the Criteria for Reporting the Development and Evaluation of Complex Interventions in health care (CReDECI 2) [24] and the Strengthening of the Reporting of Observational Studies in Epidemiology (STROBE) guidelines [25]. Following recommendations in the literature, we aimed for a sample size of 12—30 participants, prioritizing the upper limit of this range to achieve the most comprehensive and representative sample possible [[26], [27], [28]].

### 2.2 Procedure

During data collection, the nurse practitioner (NP) asked outpatient clinic patients for consent to be contacted by the coordinating researcher about study participation [29]. To maximize variability and reduce bias, random dates were selected, and all patients with appointments on those dates were approached. The NPs, informed about the study, were not involved in data collection. Patients who agreed to be contacted were listed in a designated list in the electronic patient file (Hix, Chipsoft©). The coordinating researcher then called them to explain the study and confirm their willingness to participate. Those who consented scheduled an appointment, during which they received study information, signed consent forms, used HoloVAD once, and completed a questionnaire.

The HoloVAD modules used in this study included an introduction of the HoloLens, an overview of the heart and LVAD and a module on wound care. This combination was selected to provide a balance between informative and interactive content. A complete description of HoloVAD is given in the supplemental material according to the Template for Intervention Description and Replication (TIDieR) checklist [30].

### 2.3 Data collection

Demographic characteristics and duration of LVAD implantation were collected via a questionnaire. Demographics included age, sex, country of birth and educational level. The duration of time the participant had been living with an LVAD at the time of participation was recorded, and for loved ones, their relationship with the LVAD patient was also recorded.

#### 2.3.1 Acceptability

The Dutch version of the Unified Theory of Acceptance and Use of Technology (UTAUT) was employed to measure acceptability [[31], [32], [33]]. The UTAUT comprises various domains for evaluating the acceptability of a technical device in mental health care. The questions were adapted specifically for HoloVAD, focusing on the seven domains relevant to this intervention and its outcome. In this study, outcome expectations, effort expectations, facilitating conditions, fear and knowledge were investigated [31]. The UTAUT which contains23 items, is rated on a 5-point Likert scale from 1 (totally unagreed) to 5 (totally agreed). The outcome expectations, social influence, and intention to use domains consisted of four questions ranging from 4 to 20, effort expectations, facilitation conditions and fear consisted of three items ranging from 3 to 15 and knowledge consisted of two items ranging from 2 to 10. Higher scores indicate greater feasibility for the domains.

#### 2.3.2 Usability

Usability was assessed using the System Usability Scale (SUS), which consists of a ten-item questionnaire. The SUS is a quick and effective tool for measuring the usability of technical product, such as HoloVAD and the items have been adapted for the HoloVAD [[34], [35], [36]]. Answers were rated on a 5-point Likert scale from ‘strongly disagree’ [1] to ‘strongly agree’ [5], after which a SUS score was calculated ranging from 0 to 100. The average SUS score is 68 and is an OK-to-good score; a score below 25 is the worst possible score, and a score of100 is the best possible score [35,36].

#### 2.3.3 Tolerability

Negative side effects, such as motion sickness and nausea, are occasionally experienced with the use of HMDs [37]. Tolerability refers to these side effects of the HoloLens and was assessed using the Virtual Reality Sickness Questionnaire (VRSQ) [38]. The VRSQ has two components measuring oculomotor and disorientation, both of which demonstrate good reliability (oculomotor α = 0.847, disorientation α = 0.886). Items 1—4 belong to the oculomotor component, and Items 5—9 belong to the disorientation component. Nausea, not covered by the VRSQ, is known as a possible effect of virtual reality sickness and therefore was included in the VRSQ with one additional item [39,40]. The responses for all 10 items were rated on a 4-point Likert scale, ranging from 0 (none) to 3 (severe). The total score ranges from 0 to 30, with higher scores indicating greater virtual reality sickness.

To allow participants to share additional thoughts or experiences related to HoloVAD that may not have been addressed by other questions and to gather feedback on the content of the modules, additional questions were developed by the research team. Two questions asked whether the ‘virtual instruction’ was completed or interrupted and, if so, why. Another two questions asked whether participants felt frustrated at any time and, if so, why. Three open-ended questions asked if participants found the given information clear and why, if they had any tips to improve the ‘virtual instruction’ and to describe the ‘virtual instruction’ in a few key words.

### 2.4 Statistical analysis

All analyses were performed using the 28th edition of the IBM Statistical Package for Scientific Solutions (SPSS). Demographics were analyzed by employing descriptive statistics.

Continuous variables were explored for a normal distribution through histograms. Normally distributed variables are described as the mean and standard deviation (SD) and were compared with a 2-sample independent *t*-test or as the median [quartile range (QR)] for nonnormally distributed variables and compared with a Mann—Whitney *U* test.

Categorical variables are presented as frequencies and percentages and were compared using a Pearson chi-square test when they met the requirements; otherwise, they were compared using Fisher's exact test or the Fisher-Freeman-Halton exact test when a table lager than a 2 × 2 cross table was used.

The UTAUT items were reversed if they had reversed scores, and scores were determined per domain by summing and dividing by the number of items. Acceptability was categorized as high (score > 4), moderate (score 3—4), or low (score < 3). The SUS-scores range from 0 to 100, with an average of 68. Every item ranges from 0 to 4. The score of the uneven items is the scale position minus 1, and the score of the even items is 5 minus the scale position. Finally, the sum of the scores is multiplied by 2.5 to obtain the overall SUS score. A score of >80.3 was interpreted as excellent, 68—80.2 as good, 68 as okay, 51–68 as poor and < 51 awful [35]. Each item of the VRSQ is summed and analyzed. A score of 0—1 was considered low, a score of 1—2 was considered moderate, and a score of 2—3 was considered high. The total VRSQ score was calculated across all ten items.

#### 2.4.1 Qualitative collected data were analyzed collaboratively and thematically organized

All the data collected from the study participants were compared with applicable tests to assess differences in feasibility and to identify these differences. The remaining questions covered the meaning and interpretation of the analysis and were analyzed and clustered by the research team.

### 2.5 Ethical issues

This study adhered to the latest Declaration of Helsinki and followed the code of conduct for healthcare research and the Medical Research Involving Human Subjects Act (WMO), as well as the European General Data Protection Regulation [[41], [42], [43]]. It was designed as non-WMO research since participants were not subjected to medical procedures or behavior [42]. The study was reviewed by the Medical Ethics Committee of the UMCU. The sampling and all communication with participants were performed in compliance with the General Data Protection Regulation (GDPR) [44].

## 3 Results

### 3.1 Participants

Patients were approached by the NP between February 2024 and May 2024 (*N* = 50). Six of the patients were unreachable, and four patients declined to participate. In total, forty patients were asked to participate after being informed. Fourteen patients were not included because of scheduling conflicts (*N* = 9), a patient was excluded because of an injury between the time of being approached and the appointment (*N* = 1), two patients were sick at the time of the appointment (*N* = 2), and two patients ultimately did not want to participate in the study (N = 2). Twenty-six patients were included in the final sample— nineteen who brought a loved one —for a total of 45 participants. The demographics of the study participants are presented in Table 1.

**Table 1:** Demographic variables of study participants in feasibility study.

Variable	Total *N* = 45	Patient *N* = 26	Loved ones *N* = 19	P-value
Duration of LVAD mean (SD)		37.0 [15.3–65.8]		
Age, median [QR]	59.0 [41.0–65.0]^a^	57.0 [40.0–65.0]^b^	61.0 [52.0–66.0]^b^	0.388^a^
Gender *n (%)*				0.202^a^
Women	21 (46,7)	10 (38,5)	11 (57,9)	
Men	24 (53,3)	16 (61,5)	8 (42,1)	
Birth country *n* (%)				1.000^b^
The Netherlands (%)	43 (95,6)	25 (96.2)	18(94,7)	
Different	2 (4,4)	1 (3.8)	1 (5,2)	
Education level *n* (%)				0.197^c^
Lower education	24 (53,3)	14 (53.8)	10 (52.6)	
Intermediate education	5 (11,1)	1 (3.8)	4 (21.1)	
Tertiary education	16 (35,6)	11 (42.3)	5 (26.3)	

Abbreviations: LVAD = Left ventricular assistant device, SD = Standard Deviation, QR = Quartile Range.

a Mann—Whitney U test.

b a Fisher Exact test because data does not meet the criteria for a Chi-square test.

c a Fisher—Freeman—Halton Exact Test because data does not meet the criteria for a Chi-square test and because the table is larger than 2 × 2.

### 3.2 Demographic characteristics

The average age of the patients was 57.0 years; 62 % were male (*N* = 16), the average duration of having an LVAD was 37 months (range 5—108 months), and 96 % were born in the Netherlands (*N* = 25). The average age of the loved ones was 61.0 years; 58 % were female (*N* = 11), and 95 % were born in the Netherlands (*N* = 18) (Table 1). Only men and women participated in the study; therefore, the other answer options were excluded from the calculations. No significant differences were found in the demographic characteristics of the study participants (Table 1).

### 3.3 Acceptability

A score of 4 or higher indicated that the outcome expectations, effort expectations, social influence, intention to use, facilitating conditions and fear all serve as positive determinants of acceptability for the study population, as shown in Table 2. Only the acceptance of domain knowledge is neutral for both groups. There was a significant difference in the outcome expectations for the study population. In all the other domains, no significant difference was found. Given these results, HoloVAD is considered acceptable for the study population.

**Table 2:** Acceptability of HoloVAD, measured by the unified theory of acceptance and use of technology for patients and loved ones.

	Patients	Loved ones	Total	P—value^a^
Variable	Median [QR]	Median [QR]	Median [QR]	
Outcome expectations	4.1 [3.5–4.8]	4.8 [4.2–5.0]	4.5 [4.0–5.0]	0.016
Effort expectations	4.7 [3.6–5.0]	4.7 [3.6–5.0]	4.7 [3.7–5.0]	0.683
Social influence	4.5 [3.9–4.8]	4.3 [3.8–5.0]	4.5 [3.9–5.0]	0.888
Intention to use	4.6 [4.1–5.0]	4.5 [4.0–5.0]	4.5 [4.0–5.0]	0.972
Facilitating conditions	4.6 [4.0–5.0]	5.0 [4.3–5.0]	4.7 [4.3–5.0]	0.157
Fear	5.0 [3.6–5.0]	4.7 [3.3–5.0]	5.0 [3.5–5.0]	0.487
Knowledge	4.0 [2.9–4.5]	4.0 [3.0–4.5]	4.0 [3.0–4.5]	0.787

Abbreviations: QR = Quartile Range.

a Mann—Whitney U test.

### 3.4 Usability

The overall usability score of the SUS was 82.5, as was the usability score for both groups (Table 3). This result gives the usability an A grade and ‘excellent’ rating.

**Table 3:** Usability evaluation of HoloVAD by the System Usability Scale for patients and loved ones.

	Patients Median [QR]	Loved ones Median [QR]	Total	P-value^a^
SUS score	82.5 [61.9–90.6]	82.5 [72.5–90.0]	82.5 [70.0–90.0]	0.827

Abbreviations: QR = Quartile Range.

a Mann-Whitney *U* test.

An ‘excellent’ SUS score for the study population and a score higher than 68 indicate that the HoloVAD is assessed as usable for both groups.

### 3.5 Tolerability

The total VRSQ score is 0.0, which is remarkably low (Table 4). for the oculomotor component contributed the most to the study population. General discomfort (*n* = 6) and difficulty focusing (*n* = 5) were most reported by patients. For loved ones, general discomfort (*n* = 3), difficulty focusing (n = 3), and fullness of the head (n = 3) were reported. Notably, no participants presented nausea (*n* = 0) or dizziness (with eyes closed) (n = 0). Considering these results, HoloVAD is highly tolerable by the study population.

**Table 4:** Evaluation of the tolerability of HoloVAD with the Virtual Reality Sickness Questionnaire of patients and loved ones.

	Patients	Loved ones	Total	P—value^a^
	Median [QR]	Median [QR]	Median [QR]	
Oculomotor	0.0 [0.0–0.3]	0.0 [0.0–0.3]	0.0 [0.0–0.3]	0.710
Construct	0.0 [0.0–0.0]	0.0 [0.0–0.2]	0.0 [0.0–0.1]	0.417
Total VRSQ	0.0 [0.0–0.1]	0.0 [0.0–0.2]	0.0 [0.0–0.1]	0.946

QR = Quartile Range.

a Mann-Whitney U test.

In the additional questions, participants described their experience with HoloVAD Analysis of participants' responses to the additional questions revealed three main themes characterizing their experience with HoloVAD: realism, positive learning, and distance (Table 5). Three participants, two patients and one loved one, were not able to finish the modules. Because all participants were almost finished with the last module, the collected data are included.

**Table 5:** Description and input for HoloVAD of patients and loved ones.

Question	Theme	Participants response
Can you indicate in a few key words (max 5) how you have experienced the ‘virtual instruction’?	Realistic	Clear, realistically, familiar, nice experience, nice
	Positive learning	Enlightening, exciting, educational, useful, innovative, interesting, interactive
	Distance	Lightly disoriented, tensive and impersonal
Do you have any tips to improve the ‘virtual instruction’?	Technology	Improving the reaction of the device, screen size smaller, better reaction with the gauze
	Tailored	Pauses in the instruction, option to ask questions, slower explanation, more adequate instructions of the wound care, modules more extensive, option to free practice

All participants except one patient (*N* = 44), indicated that the given information in the modules was clear. The reason given for the unclearness was that ‘the wound care appeared messy.’ Participants recommended technical improvement and options for a tailored experience (Table 5).

## 4 Discussion

This feasibility study assessed the acceptability, usability, and tolerability of HoloVAD as a novel method of patient education for patients with an LVAD and their loved ones yielding positive outcomes. Furthermore, the results highlight areas for improvement and provide considerations for the continued development of HoloVAD.

The results of our study necessitate further clarification. First, the acceptability of the UTAUT demonstrated positive outcomes with the exception of domain knowledge, which was rated as neutral. Two questions were asked in this domain; *‘I have a clear idea of what to expect from the HoloLens for getting information’* and ‘*I already have some knowledge about the information provided by the HoloLens’. Given that* this is a new experience for many participants, it is reasonable that participants do not have full knowledge of HoloVAD [45]. These results suggest that good guidance and instructions should be considered in the further implementation of HoloVAD.

Second, questions one and four of the SUS questionnaire about usability received the lowest scores. Question one is *“I think I would like to go through the ‘virtual instruction’ more often”,* and question four is ‘*I need technical support to use the ‘virtual instruction’*. This is logical given that this study population already possesses the knowledge and skills required for managing an LVAD and may not feel the need to review virtual instructions frequently. Prior to an LVAD implantation, patients have less experience; therefore, we expect that these patients would have a greater need to engage with ‘virtual instruction’ more frequently, which would likely result in better usability outcomes [2,5,8]. In the context of question four, because HoloVAD represents a pioneering technique, it is largely unfamiliar to most participants. The participants in this study described the use of the HoloLens as intensive. Therefore, adequate guidance and technical support should be prioritized in the further development and implementation of HoloVAD, particularly for more vulnerable groups, such as patients prior to LVAD implantation.

Third, regarding tolerability, the results show that both groups have high tolerability. This is consistent with other research that shows that, compared to VR, virtual reality sickness in MR is minimal or even negligible [46]. More patients scored higher on the VRSQ than their loved ones did. This suggests that, although tolerability was minimal, patients presented more virtual reality sickness than their loved ones did. Notably, all participants in the patient sample already had an LVAD and were thus more vulnerable because of their condition. Given that patients prior to LVAD implantation are even more vulnerable and have a lower QoL, this factor should still be monitored when HoloVAD is implemented [5].

As demonstrated by Wake et al. (2019), patient education with 3D models and augmented reality enhances patients' understanding of their anatomy and surgical procedures. This study revealed that only 3D models allowed for enhanced insights into the underlying anatomy and comprehension of the true size of the relevant organs and tactile feedback in addition to spatial comprehension [47]. In our study, tactical feedback was not possible; in the patients' comments, this was expressed as a desired feature. Therefore, incorporating effective tactical feedback and customizable options should be considered in the further development of HoloVAD to enhance its feasibility and effectiveness in patient education.

We identified different studies in which patient education with a HoloLens was used for different patient groups and treatments. Studies have shown that MR is a promising and helpful tool for patient education in different patient groups and can help improve the quality of patient care [48,49]. This feasibility study yields similar results regarding the feasibility of HoloVAD; however, it does not provide insight into the educational effect of HoloVAD.

Patient education using MR is a profoundly new field, with many studies still needed to further explore its potential. This study significantly contributes to the exploration of this emerging field, underscoring its importance. Furthermore, it contributes to the advancement of patient education in the field of MR.

To the best of our knowledge, this is the first study assessing the feasibility of a new patient education tool using MR for this study population. The strengths of this study include determining the feasibility of HoloVAD in a clinical setting guided by the MRC framework. This provides a reliable and credible approach to the development of HoloVAD [23]. The HoloVAD tool itself is acceptable, usable, and tolerable.

All the data collected during the study were gathered by the same researcher to minimize researcher bias, and selection bias was kept to a minimum throughout the recruitment process. These factors further strengthen the validity of this study.

A limitation of this study is that two UTAUT versions, one for end users and one for professionals, were combined. Different validated questionnaires, such as the Technology Acceptance Model (TAM), could be used to determine acceptability [50]. Although including the experiences and perspectives of the participants of HoloVAD was considered, the UTAUT was chosen to provide a wider view of acceptability. The selected questions of the two UTAUT versions were agreed upon by three researchers who considered them to be useful and applicable for this complex intervention and this patient sample.

Another limitation is that the participants included in this study were self-selected, being both interested in and enthusiastic about participating. This should be considered, as it is anticipated that the feasibility may be lower for a broader population. Furthermore, limited demographic data were collected. Neither birth country nor education level was thoroughly assessed. Moreover, data were collected only in post operative patients. Both factors limit the generalizability of the participants to the broader population. In future research, more demographic data and data in the preoperative patients should be collected to increase the generalizability of the results.

### 4.1 Innovation

An LVAD is life-changing and has a high risk of complication. HoloVAD is innovative because it integrates visualization and interaction through mixed reality, enabling a deeper understanding of life with an LVAD. This study highlights the feasibility of HoloVAD as a novel educational tool for patients and their loved ones.

Based on the findings of this study, the next step in the development of this complex intervention, HoloVAD, should be taken to further improve. In accordance with the MRC framework, a pilot phase should be developed [23]. Implementation for the study population prior to receiving an LVAD should be integrated into their care plan, with careful consideration of the patients' clinical status. Additionally, we recommend collecting more detailed demographic data in the pilot study.

### 4.2 Conclusion

In conclusion, HoloVAD is feasible, defined as acceptable, usable, and tolerable. It is a promising and helpful new education tool for patients with an LVAD and their loved ones. It gives patients the potential to engage in a preimplantation experience of life with an LVAD that, when further developed, could offer numerous benefits for the quality of patient care.

## CRediT authorship contribution statement

**Sofie E. Bresser:** Writing – review & editing, Writing – original draft, Validation, Project administration, Methodology, Investigation, Formal analysis, Data curation, Conceptualization. **Linda M. de Heer:** Writing – review & editing, Validation, Supervision, Methodology, Funding acquisition, Conceptualization. **Irene Louwerse-van Kan:** Writing – review & editing, Investigation, Conceptualization. **Faiz Ramjankhan:** Writing – review & editing. **Manon G. van der Meer:** Writing – review & editing. **Saskia W.M. Weldam:** Writing – review & editing, Validation, Supervision, Methodology, Data curation, Conceptualization.

## Declaration of competing interest

The authors declare that they have no known competing financial interests or personal relationships that could have appeared to influence the work reported in this paper.

## Appendix A Supplementary data

Supplementary material: TIDieR checklist HoloVAD Image 1
