# Supplementary material for: HoloVAD; A feasibility study of a new patient education tool for patients with a left ventricular assist device
Source: PEC Innov. 2025 Dec 6;8:100450. doi: 10.1016/j.pecinn.2025.100450 (PMC12757618; doi:10.1016/j.pecinn.2025.100450)
Supplement: Supplementary file 1 — Supplementary material: TIDieR checklist HoloVAD [file mmc1.docx]

**Supplemental material**

**Template for Intervention Description and Replication Checklist** (29)

| Item number | Item |  | Where |
| --- | --- | --- | --- |
| 1. | Brief name | The HoloVAD, patient education tool for living with a Left Ventricular Assist Device (LVAD) | Title |
| 2. | Why | Effective patient education should consist of multimodal education and needs to include cognitive and affective learning (10,13–15). It is integral to healthcare quality and must be patient centred, straightforward, and multimodal (10). Multimodal education engages multiple senses during the learning process and improves long-term and working memory (11,12). Effective education requires stimulating both cognitive and affective learning: cognitive learning involves gathering information and knowledge, while affective learning involves attitudes, satisfaction, emotional well-being, and the learners’ interests and motivation (13,14). The HoloVAD is developed with addressing these learning theories by the use of mixed reality (MR) to provide vital knowledge about living with an LVAD. Education about the expectations of living with an LVAD for both patients and their loved ones, can help manage expectations and improve coping (5,8). Therefore, effective good quality patient education is important for LVAD patients, and their loved ones and further research is required to develop interventions, particularly educational ones, for better adaptation for LVAD patients (5,8). | 1. Introduction |
| 3. | What; materials | The HoloVAD is made for a HoloLens, a mixed reality head-mounted display (HMDs). A HoloLens overlays virtual objects onto the real world (17,18). Mixed reality enriches the real world with interactive virtual data, anchored independently of head movement (16). The HoloLens offers a more immersive experience compared to earlier HMDs and should be considered as a designated HMD device in studies (16). | 1. Introduction |
| 4. | What; procedures | After the given information the participants applied the headset and started the first module. The three HoloVAD modules included an introduction of the HoloLens, an overview of the heart and LVAD and a module on wound care. This combination was selected to provide a balance between informative and interactive content. | 2.3 Procedure |
| 5. | Who provided | The nurse specialists where given information about the intention, use and content of the HoloVAD. The coordinating researcher provided the use of the HoloVAD. The researcher practiced the use of the HoloLens and content of the HoloVAD modules. The researcher provided instructions to participants for the use of the headset. The researcher was in the room while the participant used the HoloVAD. | 2.3 Procedure |
| 6. | How | All participants used the HoloVAD individually or tighter with their loved one during a face-to-face meeting. During the use of the HoloVAD participants could ask questions. | 2.3 Procedure |
| 7. | Where | The HoloVAD was used in a private room on the outpatient’s clinic of the University Medical Centre Utrecht. | 2.3 Procedure |
| 8. | When and how much | Patients with an LVAD, and their loved ones, were seen once at the outpatient’s clinic before or after their scheduled appointment. | 2.3 Procedure |
| 9. | Tailoring | All information provided by their nurse specialist or the coordinating researcher identical for all participants. Furthermore, all participants followed the same the modules of the HoloVAD. However, if participants had questions these were answered by patient’s needs. |  |
| 10. | Modifications | The HoloVAD was not modified during this research |  |
